# InChI: connecting and navigating chemistry

**DOI:** 10.1186/1758-2946-4-33

**Published:** 2012-12-13

**Authors:** Antony J Williams

**Affiliations:** 1 Royal Society of Chemistry, US Office, 904 Tamaras Circle, Wake Forest, NC, 27587, USA

## Abstract

The International Chemical Identifier (InChI) has had a dramatic impact on providing a means by which to deduplicate, validate and link together chemical compounds and related information across databases. Its influence has been especially valuable as the internet has exploded in terms of the amount of chemistry related information available online. This thematic issue aggregates a number of contributions demonstrating the value of InChI as an enabling technology in the world of cheminformatics and its continuing value for linking chemistry data.

## Editorial

To state that the InChI has had a dramatic impact on my career would be an understatement. I am one of the founders of ChemSpider[1,2], an online public compound database used by thousands of chemists every day, and it was developed with InChI as one of underpinning technologies. It is definitely true to declare that without InChI as an enabling technology ChemSpider is unlikely to have progressed at the pace it did, it would be a lot less functional in terms of its capabilities to connect to chemistry on the internet and would not hold its present prominence as one of the primary community resources for chemists online. ChemSpider became such a success as a “hobby project” that it was acquired by the Royal Society of Chemistry where our growing team of cheminformatics experts develop and support new capabilities including the development of a reactions database, a micro-publishing platform (ChemSpider SyntheticPages[3]) and the integration of chemistry data to our publications. InChI underlies and therefore has an influence on *all* of these projects.

I am fortunate to have been at some of the earliest meetings where the concept of a new chemical identifier, and its potential value to the developing environment of the internet, was touted. I clearly recall the excitement and support of a number of the internet chemist evangelists balanced against the naysayers who considered the issue of “chemical identifiers” fully solved. Looking back in retrospect is an amusing exercise since despite being one of the earliest supporters, InChI has achieved far more than I ever imagined, a lot of this supported, of course, by the incredible growth and power delivered via internet technologies. InChI has caused a disruptive momentum in terms of the flow of chemistry data onto the internet and, coupled with the open science movement of Open Data, Open Source, Open Standards and Open Access, has become one of the foundation technologies enabling chemistry on the internet. That said, the majority of practicing chemists still do not know what an InChI is (perhaps they do not need to?).

In 2012 my collaborator and friend Alex Tropsha (UNC, Chapel Hill) and I hosted a symposium at the American Chemical Society meeting in San Diego. The symposium hosted a dozen presentations and was dedicated to providing an overview of the impact that the InChI has had on cheminformatics since its inception. By the end of the day it was obvious that InChI has been embraced not only by cheminformatians but also by publishers, by public compound database hosts, by scientists integrating chemical and biological data, by commercial software companies, by pharmaceutical companies and, whether they know they are using InChI or not, by chemists of all types around the world.

This thematic issue is a natural follow on from that gathering. It includes contributions from a number of the speakers and organizations represented at the InChI Symposium. However, in developing this collection of articles I have cast the net broader, inviting contributions from other groups. The papers collected here come from academia, government labs, publishers, commercial software vendors, pharmaceutical companies and those involved with the development of InChI at the front line. At the time of writing this introduction not all papers are submitted, but the list of committed articles is sure to make for stimulating reading. In any case, the thematic issue will remain open for future submissions associated with InChI as the technology expands in capability and applications.

Chemistry is itself complex and InChI support for it is far from complete yet the impact of the first series of releases has been to enable a dramatic improvement in handling chemistry. The future of InChI is assured, certainly for the mid-term. Predicting the long term in our domain is close to impossible! As discussed in the papers within this thematic issue work is already underway in a number of areas including the reaction InChI, improving the handling of organometallics and inorganic compounds, handling polymers and Markush structures. The reach of the InChI identifier will continue to expand, (why not become a standard for large biomolecules?). We, as a community, owe a great debt of gratitude to the vision of the few individuals who initiated the development of InChI, the very small team of developers who did the heavy lifting of developing the code and to the cheminformaticians who actively embraced InChI even in its earliest variants. Chemists around the world are benefiting from InChI as a standard that has been embraced by those who need it and at the same time it offers a valuable foundation for the creation of chemical compound databases.

I am grateful to all the authors for their contributions to this stimulating set of articles, demonstrating the impact of InChI on the world of chemistry, cheminformatics and linking of chemistry data. We have traveled a long way in a short time, InChI by InChI.

## Competing interests

The author is the host for the ChemSpider database and related projects at the Royal Society of Chemistry.
